# Supplementary material for: Feasibility of a new multifactorial fall prevention assessment and personalized intervention among older people recently discharged from the emergency department
Source: PLoS One. 2022 Jun 9;17(6):e0268682. doi: 10.1371/journal.pone.0268682 (PMC9182319; doi:10.1371/journal.pone.0268682)
Supplement: S1 Appendix — (DOCX) [file pone.0268682.s001.docx]

S1 table 1: Description of risks identified through Client Assessment Protocols in the interRAI-HC assessment instrument [18].

**Risk category Client Assessment Protocol Description**

Clinical status Cardio-respiratory health Identifies people who suffer from cardiorespiratory conditions (e.g., chest pain, shortness of breath, irregular pulse, dizziness).

Dehydration Identifies people who show signs of dehydration or a disrupted fluid balance.

Delirium Identifies people who show active symptoms of delirium (e.g., easily distracted, unstable consciousness, acute cognitive decline).

Faecal incontinence Identifies people who suffer from faecal incontinence, and for whom bowel function could be improved or decline could be prevented.

Nutrition Identifies people who show signs of malnutrition (BMI<21)

Pain Identifies people who suffer from pain on a daily basis.

Pressure ulcer Identifies people with pressure ulcers or people who are at risk of developing pressure ulcers.

Urinary Incontinence Identifies people who suffer from urinary incontinence, and for whom bladder function could be improved or decline could be prevented.

Functioning Activities of Daily Living (ADL) Identifies people who are at risk of a decreasing ability to independently perform ADL or for whom ADL abilities could be improved.

Cognitive functioning Identifies people with no or only mild cognitive impairments, who show at least two risk factors for cognitive decline (e.g., dementia, communicative problems, disorientation, confusion, restlessness).

Communication Identifies people with communicative problems (i.e., problems with expressing themselves and/or understanding others) that could be improved, or for whom further decline could be prevented.

Falls Identifies people who experienced one or more fall incidents during the past 90 days, who are at risk of experiencing another fall incident.

Instrumental Activities of Daily Living (IADL) Identifies people for whom IADL ability could be improved and who have no or only mild cognitive impairments.

Mood Identifies people who are at risk of developing a depressive disorder.

Risk of institutionalization Identifies people who are at high risk of admittance to an institutional care facility in the following months.

Lifestyle and physical environment

Behaviour Identifies people who have shown behavioural problems (e.g., wandering, verbal or physical violence, socially inappropriate behaviour) during the last three days.

Physical activity Identifies people with < 2 hours of physical activity in the last 3 days, who do not have (physical) limitations to be more physically active.

Smoking and drinking Identifies people who smoke on a daily basis and consume alcohol on an incidental to regular basis.

Social and physical environment

Abusive relationship Identifies people who are at risk of abuse, based on one or more indicators of abuse (e.g., scared of relative or caregiver, showing signs of neglect or maltreatment) combined with one or more stress factors (e.g., BMI< 18, depression, social isolation, upset caregiver)

Home environment Identifies people who show at least two signs of frailty (e.g., unable to climb stairs, unstable gait, poor or unstable health, depressive symptoms, hallucinations) and live in a problematic home environment (e.g., dilapidation, filth, problems with lighting, carpets, kitchen, bathroom, access to rooms).

Informal care Identifies people who need help with at least one IADL area and who have a brittle informal support network (i.e., at least two of the following: spend most of their time alone, live alone, have no primary informal caregiver).

Social function Identifies people who report feeling lonely or who show no or declined social involvement in their community.

S2 table 2: results of CAPs of the intervention group

Clinical status

Cardio-respiratory health

    Not triggered n (%)       10 (50%)

    Triggered n (%)  10 (50%)

Dehydration

Not triggered n (%)            17 (85%)

    Triggered n (%)                      3 (15%)

Delirium

     Not triggered  n (%)                        19 (95%)

     Triggered  n (%)                           1 (5%)

Faecal incontinence

Not triggered  n (%)                         16 (80%)

     Triggered  improvement possible n (%)          1 (5%)

Triggered improvement not possible n (%)  3 (15%)

Nutrition

  Not triggered n (%)                  13 (65%)

    Triggered risk BMI 19-22 n (%)  4 (20%)

    Triggered high risk BMI <19  n (%)          3 (15%)

Pain

  Not triggered n (%)         7 (35%)

    Triggered-medium priority   n (%)          9 (45%)

    Triggered-high priority n (%)           4 (20%)

Pressure ulcer Not triggered      n (%)          17 (85%)

    Triggered   n (%)                         3 (15%)

Urinary Incontinence

   Missing values  n (%)                    5 (25%)

Not triggered- poor decision making at baseline n (%)    1 (5%)

    Not triggered- continent at baseline n (%)    10 (50%)

    Triggered- prevent decline n (%)            2 (10%)

    Triggered - facilitate improvement n (%)    2 (10%)

Functioning

Activities of daily life

    Not triggered  n (%)                   7 (35%)

    Triggered- prevent decline n (%)            10 (50%)

    Triggered - facilitate improvement n (%)   3 (15%)

Cognitive functioning

    Not triggered n (%)                    5 (25%)

   Triggered- prevent decline     15 (75%)

Communication

    Not triggered n (%)  18 (90%)

Triggered - potential for improvement  n (%)   0 (0%) Triggered - risk of decline n (%)             2 (10%)

Falls

    Triggered - low risk n (%)                 18 (90%)

    Triggered - high risk  n (%)              2 (10%)

Instrumental Activities of Daily Living (IADL)

Not triggered  n (%)    13 (65%)

    Triggered   n (%)                7 (35%)

Mood

    Not triggered   n (%)      11 (55%)

    Triggered - low risk  n (%)           6 (30%)

    Triggered - high risk n (%)          3 ( 15%)

Risk of institutionalization

Missing n (%) 3 (15%)

Not triggered n (%) 13 (65%)

Triggered n (%) 4 (20%)

Lifestyle and physical environment

Behaviour Not triggered  n (%)              19 (95%)

    Triggered prevent almost daily behavior n (%)      1 (5%)

Physical Activity Promotion

    Not triggered n (%)    13 (65%)

Triggered  n (%)             7 (35%)

Smoking and Drinking

    Not triggered  n (%)     16 (80%)

    Triggered n (%)                    4 (20%)

Social and physical environment

Abusive relationship

Not triggered n (%) 7(%)

Triggered- medium risk. n (%) 12 (60%)

Triggered- high risk n (%) 1 (5%)

Home environment

Not triggered n (%)           19 (95%)

    Triggered n (%)                   1 (5%)

Informal care

Not triggered n (%)             11 (55%)

    Triggered n (%)                   9 (45%)

Social function

Not triggered n (%) 16 (80%)

Triggered n (%) 4 (20%)

S3 Table 3: quotes from the focus group discussion

| **Subject** | **barriers quotes** | **Facilitators quotes** |
| --- | --- | --- |
| Communication between healtcare professionals | “The assessment transmission by e-mail from assessor to GP or nurse practitioner is hard to interpret” (nurse practitioner) | “Use siilo application for communication” (research team) |
|  | “Communication not compelling enough and needs more explanation”(assessor 1) |  |
|  | “Connection between ED and screener must stay” (assessor 2). |  |
| Motivation of the frail elderly | “Problem is often downplayed” (healthcare innovator) | “it is important to convince the elderly of the important of fall prevention directly after the fall” (GP) |
|  | “The non-motivated people are also often the people who fall a lot, it is important to help here” (GP) | “At ED, necessity of fall prevention became clear” (assessor 2). |
|  |  | “motivational interviewing as instrument for better motivation” (assessor 2). |
|  |  | “A known person, gaining trust” (GP) |
|  | “The term fall prevention deters people (…..) ”(assessor 2) | “(….) staying independent attracts people more”(assessor 2) |
|  |  | “Add short physical test as motivator to start interventions“(assessor 1). |
| interRAI-HC assessment | “in my opinion the interRAI-HC is too extensive and difficult to interpret” (nurse practitioner) | “the interRAI-HC is a wide measuring instrument and a good and fast way of insight“ (assessor 2) |
|  | “Takes a few times before you realize how to properly conduct the screening” (researcher 2) | “first consult the nurse practitioner for some background of the patient and discus if the interRAI-HC assessment is helpful“(nurse practitioner & assessor 2). |
| ED | “ED nurses don’t have the time for inclusion” (research team) | “In the future it must be in the duties of the ED nurses” (research team) |
|  |  | “Because people are in the hospital, they are more likely to enter the care process” (assessor 1) |

*There were two assessor’s participating the focus group, assessor 1 and assessor 2.
