# Supplementary material for: Feasibility of a new multifactorial fall prevention assessment and personalized intervention among older people recently discharged from the emergency department
Source: PLoS One. 2022 Jun 9;17(6):e0268682. doi: 10.1371/journal.pone.0268682 (PMC9182319; doi:10.1371/journal.pone.0268682)
Supplement: S1 File — (DOCX) [file pone.0268682.s002.docx]

Research protocol

**General information.**

| **Title** | Feasibility study into the development of an RCT on the prevention of re-fall after a presentation with a fall-related incident in the emergency department without admission.**- Project Draaideur -** |
| --- | --- |
| **Date** | 26 november 2018 |
| **Version nummer** | Version 2 |
| **submitter** | E.S van den Ende MD, PhD student acute health care |
| **Coördinering researcher** | Drs. E.S. van den Ende  (Amsterdam UMC, location VUmc, acute health care)  [e.vandenende@vumc.nl](mailto:e.vandenende@vumc.nl)  Prof. Dr. N. van der Velde  (Amsterdam UMC, location AMC, internal medicine, section elderly health care)  n.vandervelde@amc.uva.nl  E. Geleijn  (Amsterdam UMC, location VUmc, rehabilitation unit, Fysiotherapie)  [e.geleijn@vumc.nl](mailto:e.geleijn@vumc.nl)  Prof. Dr. H.P.J. van Hout  (VU university Amsterdam, elderly health care)  T. Samson  Physiotherapie, masterstudent Evidence Based Practice UVA  tamarsamson@hotmail.com |
| **Lead researcher** | Dr. P.W.B. Nanayakkara  p.nanayakkara@vumc.nl, VU Medisch Centrum, De Boelelaan 1117, 1081 HV, Amsterdam. Kamer 1D053 |

**Research information**

| **Rationale** | The number of elderly people is increasing and with it the number of frail elderly people. The term frail elderly is used for people over 65 who have an increased risk of hospitalization and increased dependence on the basis of illness, reduced physical, cognitive and/or social functioning. The societal challenge is to support these vulnerable elderly people in maintaining their vitality and thus quality of life as much as possible [1-4]. Falls are a threat to the quality of life of the elderly. A fall is defined as “an unintentional change of body position, resulting in a landing on the ground or another lower level”. Fall incidence increases with age. International research shows that approximately twenty to thirty percent of people aged 65 and older living independently fall at least once a year, fifteen percent fall at least twice or more per year [5]. Nationally, approximately 90,000 elderly people present themselves each year in the emergency department (ED) of a hospital in connection with a fall-related incident. More than 3000 elderly people die as a result of this fall, which makes fall incidents 8th in the list of causes of death in the Netherlands [7,8] The number of fatal falls has increased by 40% in the past five years [22].  In Amsterdam, 38,000 inhabitants fell at least once in the past year (AGM, reference year 2016). Every year, approximately 1,500 elderly people over the age of 70 present themselves to the emergency department of VUmc after a fall-related incident (figures 2017). About a third of patients are hospitalized. Of the patients who can go home, more than a third are seen again in the ED within 1 month, of which 20% again with a fall-related problem [8]. Nationally, the number of re-presentations is 25%. These patients are subsequently responsible for half of the total number of A&E presentations [9].  In addition to increased mortality, falls also lead to a decrease in quality of life and an increase in healthcare costs. In the trauma region of the North-West of the Netherlands, for example, a 2.4% increase in hospital admissions was seen in the past 3 years among frail elderly [10]. Much research has already been done into the epidemiology of falls, the risks of their occurrence and interventions to prevent them. There are also many smaller and larger initiatives aimed at reducing it. These are often based on mapping out risk factors such as polypharmacy, visual disturbances, reduced physical fitness, reduced intake of vitamin D and obstacles in the physical living environment of the elderly [11]. Multifactorial interventions can be aimed at these risks that demonstrably decrease the risk of falling, such as medication optimisation, exercise programmes, home modifications and the introduction of aids. The RIVM Center for Healthy Living has identified In Balans, Fallen Verleden Tijd, Otago and View on Balance as effective exercise programs for reducing the number of falls and/or fear of falling. These interventions differ in design and target group [12,13]. Moderate to good evidence of effectiveness is available in all four interventions. Most of these interventions are aimed at elderly (65+) living independently who have a fear of falling or an increased risk of falling. The emphasis in these interventions is on primary fall prevention, the prevention of falls and less on secondary fall prevention, ie the prevention of new falls in people who have already fallen one or more times. A major problem with interventions aimed at primary fall prevention is that a large proportion of elderly people without a history of falling are unwilling to participate because they do not realize that they have an increased risk of falling [14]. Another problem is that these programs address the risk factors mobility and fear of falling and are therefore not suitable as a single intervention for a patient with an injury after a fall. Multifactorial assessment and treatment should be performed in this group of patients.  Elderly people suspected of serious injury after a fall-related incident are admitted to the emergency room of a hospital. During the emergency room visit, the focus is on the analysis of the acute medical problem and little attention is paid to the cause of the fall or identifying risk factors for re-falling. The fall prevention guideline recommends a multidisciplinary assessment after a fall and regional agreements on the fall prevention care path.  Within Project Revolving Door, customized care is offered after a visit to the A&E with the aim of preventing repeated falls and the associated comorbidity. The day after the visit to the emergency room, the elderly person is called at home by an employee of the hospital concerned. In this meeting, it is offered that a specifically trained physiotherapist from the living environment makes a home visit to determine, by means of an extensive fall assessment in accordance with the national multidisciplinary guideline Prevention of falls in the elderly, which measures can be taken to limit the risk of falling again.  This can mean a package of measures from the general practitioner, home or community care, occupational therapist, dietician or clinical geriatrician or geriatric specialist, or only the use of a physical fall prevention program by a physiotherapist [16]. The targeted use of interventions on the individual needs of the elderly increases the willingness to participate and also saves costs. | |  |
| --- | --- | --- | --- |
| **Goal** | Feasibility analysis of the secondary fall prevention interventions resulting from the fall analysis according to the guideline Prevention of falls in the elderly.  Attention will be paid to the feasibility of the procedural, organizational and social aspects and the reasonably expected results of the Revolving Door Project.  The framework REAIM (Reach – Effectiveness – Adoption – Implementation – Maintenance) will be used.  1. Reach: The number of patients potentially to be included, the number of patients actually included, the number of patients who complete the entire process. The focus will also be on the number of care providers who are willing to participate in the project.  2. Effectiveness: The satisfaction of patients and caregivers with the project and the interventions. In the pilot phase, attention will also be paid to whether a trend is already visible in the decrease in the number of re-presentations in the ED.  3. Adoption. The number of institutions and care providers that are willing to participate in the before and after study will be examined.  4. Implementation: are the innovations successfully implemented? We will look at what the best form is to allow a multidisciplinary team to function around each patient.  5. Maintenance: It will be examined whether the interventions still have a positive effect on the patient after 6 months. The aim of the study is also to find a sustainable way to provide multidisciplinary care for patients after a fall. | |  |
| **Reasearch design** | | Feasibility study into the development of a fall prevention intervention study, conducted at the AUMC, location VUmc.  Patients who meet the established inclusion criteria will be asked to participate in the pilot study. A total of 50 patients will be included.  In addition to receiving an information brochure, participating patients will be screened in accordance with the Prevention of Falls in the Elderly guideline. The resulting interventions will be carried out in the following weeks.  Before the start of the study, all relevant healthcare providers in the region will have been informed about the project and will know what to do if patients are referred. Existing channels are used for communication (first line Amsterdam / Collaborative institutions health care region Amsterdam (SIGRA) / Regional Consultation Acute Care Chain (ROAZ)). General practitioners will be informed and actively involved through the Integrated Primary Care (GEZZ South). Conventional and social media will be used to publicize the project and thereby raise awareness about fall-related issues. This is in line with the campaign strategy developed by the GGD under the heading *laatjenietvallen* . There are several ways of collaborating with the GGD.  For example, first-line care providers (physiotherapists or occupational therapists, nurses or nurse practioners) who have been trained in the performance of the fall analysis in the context of the GGD project are involved in the Revolving Door project. After the analysis, they will refer the patient to other healthcare providers. This also includes the implementation of an effective fall prevention program tailored to the possibilities and needs of the patient.  Quantitative and qualitative data collection will take place in Castor (a secure digital database). Data processing and analyzes will be performed within the VUmc. | |
| **Population** | | 50 patients aged 65 years or older, presented at the emergency department of the AUMC location VUmc, with a low-energy fall-related complaint, without admission indication. | |
| **Inclusion ecriteria** | | - 65 years or older  - Living (semi) independently (i.e. in an assisted living accommodation, completely independently)  - Presented at ED with a fall-related complaint  - to be linked to a trained professional (i.e. living in the vicinity of a trained 1st line care provider, insured for the necessary care or willing to pay for it yourself) | |
| **Exclusion criteria** | | - Admission to hospital or other healthcare institution  - Impeding injury  - Unable to give informed consent  - Insufficient command of the Dutch or English language  - Patients who already participate in a fall prevention project.  - Patients living outside the Amsterdam region | |
| **Sample size** | | With the inclusion of 50 patients, we expect to be able to make a reasonable statement about the feasibility of the Revolving Door Project. | |
| **Recruitment of test subjects and course of research** | | During an emergency room visit: Patients who are suitable for inclusion will be asked to participate in the study upon presentation at the emergency room of the AUMC, location VUmc. If the patient agrees to participate, he or she will receive an information letter and informed consent form and an executive researcher will contact the patient or his/her caregiver by telephone after 24 hours.  24 hours after ED visit  During the telephone conversation, the research will be explained again and the patient will be asked to return the signed informed consent form to the VUmc. After approval, a short questionnaire will be administered (Appendix 1). After the telephone conversation, the conducting researcher contacts the nearest participating primary care provider to plan the fall analysis in the home situation.  < 72 hours after ED visit:  A trained first-line care provider (physiotherapist or occupational therapist, nurse or POH-er) visits the participant at home and performs the fall analysis of the guideline Prevention of falls in the elderly. It also determines whether additional care is required as a result of the fall analysis.  On the basis of the results of the fall analysis, the trained primary care provider draws up a treatment plan in consultation with the patient and will present it to other care providers (ie home care, POH, community team or physiotherapist) if they are already involved (preferably in a multidisciplinary team). consultation (MDO)). If other specialisms have to be involved, this will be organized by the coordinating researcher. The coordinating investigator will inform the general practitioner of the examination and the treatment plan that has been drawn up (preferably in an MDT) and, where possible, involve his/her help in coordinating the care, the additional history-taking and physical examination and blood tests if indicated.  3 months after ED visit:  Another short telephone interview will be conducted by the conducting researcher (in which the patient's experiences with regard to the study and the interventions will be discussed. There will also be a focus on fear of falling, ADL dependence and care consumption (See Appendix 1) ). | |
| **Intervention** | | - Standard care - Information folder - Physiotherapy* - Fall analysis of the guideline Prevention of falls in the elderly and the resulting interventions** - *Physiotherapy: - -Month 0-3: active training under supervision (in a group, if not fit enough then at home) - Month 3-6: independent practice / movement.   ** Possible interventions based on fall analysis:   - Sanitize medication - Adjusting physical living environment - Nutritional advice - Reduce fear of falling - Treating Visual Impairment - Determining the seriousness, nature and, if necessary, guiding the patient / caregivers - Support with self-care / admission to a healthcare institution - Opportunities for expanding social participation - Adjust footwear - (Optimise) medical treatment of fall risk-increasing co-morbidity - Identifying possible underlying acute conditions such as infections or electrolyte disturbances | |
| **Study endpoints** | | Feasibility of the procedural and organizational aspects (See also RE-AIM framework):  - Number of patients willing to participate  - Number of patients unwilling to participate and reason for this  - Number of patients who go through the entire process  - Feasibility of screening method  - Feasibility of interventions (adequate training of external professionals, patient response).  - Is the multidisciplinary nature of the study manageable (involving and motivating external professionals)  - Experience and feedback from patients and caregivers  With regard to the effect of interventions:  1. Recurrences of fall-related incidents (self-reporting via fall calendar, supported by telephone follow-up)  2. Representations in the ED after a fall-related incident within 3 months (Hospital EPD)  3. 3-month mortality (EPD via municipal population register)Difference score of fear of falling and ADL dependence, emerging from the fall analysis and telephone interviews after 72 hours and 3 months after A&E presentation | |
| **Study parameters** | | At baseline:  *Telephone interview (within 24 hours after ED visit, Appendix 1)*  *1. (Questionnaire about the situation before the incident (Patient characteristics, living conditions, healthcare consumption, fear of falling, physical functioning, quality of life, reason for falling, previous experience with falls))*  *Intervention arm: home visit by a trained first-line care provider (within 72 hours after A&E visit) for Valanalyse SafetyNL (including transfer to GP)*  *After 3 months*  *Telephone interview (Appendix 1)*  *- Qualitative (in patients in intervention cohort): Experiences with the interventions within this study. What does the patient encounter? Do things have to be different? What things are going well?*  *- Quantitative: Healthcare consumption (extramural and admissions) (medical costs), New falls, Fear of falling, Recovery of physical function (Validated IADL questionnaire), Quality of life (validated questionnaire)*  *Hospital EHR*  *1. SEH . Representations*  *2. Mortality* | |
| **Statistical analyses** | | Qualitative data will be open coded.  Statistical tests will be used for quantitative data. Correlation of numerical variables will be performed by means of a Pearson correlation analysis. Differences in continuous variables between two groups will be analyzed with a t-test. Chi-square test will be used to compare numbers between subgroups. A p value of <0.05 will be considered statistically significant. Analyzes will be performed using Statistical Package for Social Sciences for Windows (SPSS). | |
| Load for the participants | | - The patient will be interviewed twice by telephone (2x30 minutes) - Status examination The patient must be willing to participate in the personalized intervention program for 6 months (e.g. physiotherapy, consultation with an ophthalmologist) and will sometimes have to come to another location (if possible). | |
| **Risk for participants** | | No risks apply, all interventions will be started in consultation with the patient. The physical fall prevention programs that are used have proven to be safe and effective. | |
| **Benefits of participating in the study** | | Possibly positive effects on daily functioning and reduction of the risk of falling in patients in the intervention group. | |
| **Disadvantages of participating in the study** | | - The time the interviews take - The time the interventions take - Costs: All interventions, except physiotherapy, are covered by the basic insurance. However, the deductible will be used if the care cannot be provided by the GP or POH. Costs that are not covered by the basic insurance are not reimbursed. | |
| **Reimbursement for test subject** | | Patients participating in the study will not receive any financial compensation. Costs that are not covered by the basic insurance will be reimbursed. Travel costs incurred will not be reimbursed. | |
| **Administrative aspects** | | No directly identifiable patient data will be collected. Data is stored in a secure Castor digital database. The link between the study number in Castor and the names of the patients is known only to the project team. Data will be analyzed in the VUmc. | |
| **Publication Policy and Amendments** | | The aim is to process the results obtained in a medical scientific publication. | |
| **Referenties** | | **Literatuur**   1. <http://www.beteroud.nl/docs/beteroud/over-ons/Toekomstvisie-wonen-welzijn-zorg-ouderen.pdf> 2. <https://laego.nhg.org/sites/default/files/content/laego_nhg_org/uploads/visie_op_de_eerstelijnsgeneeskunde_voor_ouderen_20141.pdf> 3. <https://www.nivel.nl/sites/default/files/bestanden/Overzichtstudie-ouderen-van-de-toekomst.pdf> 4. <https://www.scp.nl/Publicaties/Alle_publicaties/Publicaties_2011/Kwetsbare_ouderen> 5. CBO. Richtlijn Preventie van valincidenten bij ouderen. Nederlandse Vereniging voor Klinische Geriatrie, 2004. 6. VeiligheidNL. Vallen 65 jaar en ouder. 2015 7. CBS statline. <http://statline.cbs.nl/StatWeb/publication/?VW=T&DM=SLNL&PA=82899NED&LA=NL> Bezocht juli 2017 8. E.J.M. Schrijver, Q. Toppinga, O.J. de Vries, M.H.H. Kramer, P.W.B. Nanayakkara. An observational cohort study on geriatric patient profile in an emergency department in the Netherlands. Neth J med. 2013; vol 71;324-30 9. <http://www.vilans.nl/docs/producten/Verbeterpakket_Valpreventie.pdf> 10. ROAZ Noord-West Nederland. Rapport patiëntstromen 2013 – 2016. Juli 2017 11. NZa, Marktscan Acute Zorg. Zomer 2017 12. <http://www.vilans.nl/docs/vilans/publicaties/haalbaarheid-van-health-impact-bond-valpreventie-ouderen.pdf> . 13. [www.veiligheid.nl/valpreventie](http://www.veiligheid.nl/valpreventie) 14. Gillespie LD, Robertson MC, Gillespie WJ, Sherrington C, Gates S, Clemson LM, Lamb SE. Interventions for preventing falls in older people living in the community.Cochrane Database Syst Rev. 2012 Sep 12;(9):CD007146 15. Akker D van den, Dehara J, Hoffman D, Jellema R, Molenaar D. Fall Intervention and the Needs of Elderly, Qualitative Study. Hogeschool van Amsterdam, Product Assignment 2016 16. <https://www.veiligheid.nl/valpreventie/trainingen> 17. Conceptrichtlijn valpreventie bij ouderen. Nederlandse Vereniging voor Klinische Geriatrie 2017 18. Matchar DB, Duncan PW, Lien CT, Ong ME, Lee M, Gao F, Sim R, Eom K. Randomized Controlled Trial of Screening, Risk Modification, and Physical Therapy to Prevent Falls Among the Elderly Recently Discharged From the Emergency Department to the Community: The Steps to Avoid Falls in the Elderly Study. Arch Phys Med Rehabil. 2017 Feb 12 19. Bergland A, Wyller TB. Risk factors for serious fall related injury in elderly women living at home. Inj Prev. 2004 Oct;10(5):308-13. 20. de Gelder J, Lucke JA, de Groot B, Fogteloo AJ, Anten S, Mesri K, Steyerberg EW, Heringhaus C, Blauw GJ, Mooijaart SP. Predicting adverse health outcomes in older emergency department patients: the APOP study. Neth J Med. 2016 Oct;74(8):342-352. 21. SIGRA. Krakende ketens in de zorg voor kwetsbare ouderen. Mei 2017 22. <http://www.ggd.amsterdam.nl/beleid-onderzoek/gezondheidsmonitors/gezond-wel/valongevallen/aanpak-valongevallen/> bezocht op juli 2017 23. https://www.cbs.nl/nl-nl/nieuws/2017/43/steeds-meer-doden-door-een-val | |
