# Supplementary material for: Feasibility of a new multifactorial fall prevention assessment and personalized intervention among older people recently discharged from the emergency department
Source: PLoS One. 2022 Jun 9;17(6):e0268682. doi: 10.1371/journal.pone.0268682 (PMC9182319; doi:10.1371/journal.pone.0268682)
Supplement: Attachment — Submitted filename: Response to Reviewers.docx [file pone.0268682.s005.docx]

March 16, 2022

Editor in Chief

Dear Editor in Chief and reviewers,

Thank you for your comments on our article entitled “Feasibility of a new multifactorial fall prevention assessment and personalized intervention among older people recently discharged from the emergency department.”.

We confirm that the required revisions have been performed. Below a point by point response to reviewers comments:

1) *We note that you have selected “Clinical Trial” as your article type. PLOS ONE requires that all clinical trials are registered in an appropriate registry*

Registered on November 8, 2019, registration number; NTR NL8142

2) Registration done retrospectively

The final conformation at the website was indeed performed a week after the start of the study. However, we were busy with the registration long before we started the study.

3) *We note that the grant information you provided in the ‘Funding Information’ and ‘Financial Disclosure’ sections do not match.*

We received the grand from ZonMW (grandnumber: 2006533, E 37500,- ) and we received an additional grand form municipal health services Amsterdam (E15000,- ).

4) *We note that you have indicated that data from this study are available upon request. PLOS only allows data to be available upon request if there are legal or ethical restrictions on sharing data publicly.*

We will submit the available data.

5) *Review Comments to the Author; Reviewer #1: This is a pilot study to evaluate feasibility. Statistics are descriptive and no formal hypothesis test can be performed. I just have minor concerns:
   Table 1.
   % for number of falls are not correct. Sum <100% and this variable should not have missing values.
   % for number of comorbidities do not seem correct. Both treat and control summed more than 100%.
   % for number of medications, more than 100%.
   IADL use dot for decimal number.*

The incorrect sum calculation in table one have been solved (see manuscript).

6) *There are many elderly fall patients in the emergency department, but the cases collected in the two groups in this paper are both small. I see that the author only collected the cases for 3 months, so I want to know why not collect more cases for a longer time? The results might be more convincing.*

The reason to only collect cases for three months is, because in this qualitative study, after three months we reached data saturation.

7) *In line 105, could you explain a low energetic fall related injury in detail? How did you evaluate that?*

Low-energy fall is defined as a result of **falling from standing height or less**, while high-energy trauma is defined as any other type of trauma (e.g. falling from height higher than standing height and motor vehicle accident). With the changing patient mix presenting at the emergency departments we see an increase influx of elderly with a low energetic fall related injury contrary to high energetic trauma related to road traffic accidents and violence. Care chain for high energetic trauma is well developed in the Netherlands while care-pathways for increasing low-energy trauma is not implemented well yet.

Please address all correspondence concerning this manuscript to me at [b.hepkema@amsterdamumc.nl](mailto:b.hepkema@amsterdamumc.nl).

Thank you for your consideration of this manuscript.

Sincerely,

B.W. Hepkema, MSc

Department of rehabilitation VUmc

Amsterdam University Medical Center (AUMC)

De Boelelaan 1117, 1081HV Amsterdam

The Netherlands
